# Treatment of multiple huge liver cysts in a hybrid operating room: a case report

**DOI:** 10.1186/s40792-021-01320-6

**Published:** 2021-10-29

**Authors:** Sho Ishikawa, Shintaro Kuroda, Keigo Chosa, Kenjiro Okada, Naoki Tanimine, Hiroyuki Tahara, Masahiro Ohira, Kentaro Ide, Tsuyoshi Kobayashi, Hideki Ohdan

**Affiliations:** 1 Department of Surgery, Kure City Medical Association Hospital, 15-24, Asahimachi, Kure-City, Hiroshima, 737-0056 Japan; 2 Department of Gastroenterological Surgery, Hiroshima University Hospital, 1-2-3 Kasumi, Minami-ku, Hiroshima, 734-8551 Japan; 3 Department of Diagnostic Radiology, Hiroshima University Hospital, Hiroshima, Japan

**Keywords:** Huge liver cysts, Hybrid operating room, Angiography, Unroofing, Hepatic cystotomy

## Abstract

**Background:**

Liver cysts are common, with most cases being asymptomatic. In symptomatic cases, the disease is amenable to treatment. However, huge or multiple liver cysts with vascular narrowing and associated systemic symptoms are extremely rare. Furthermore, the performance of a reliable and effective surgery in such cases remains a major problem. Here, we report a case of multiple giant liver cysts with impaired blood flow surgically treated in a hybrid operating room.

**Case presentation:**

A 73-year-old male presented to a previous doctor with leg edema and dyspnea on exertion; computed tomography revealed that the cause complaint was right lung and heart compression and inferior vena cava (IVC) stenosis due to huge liver cysts in the caudal lobe. The patient was referred to our hospital because of disease recurrence despite percutaneous aspiration of the cyst. Multiple liver cysts were observed in addition to the drained cysts, two of which were located on both sides of the IVC and caused IVC stenosis. We performed open surgery for the liver cysts and used the hybrid operating room for intraoperative IVC angiography and measuring the hepatic vein and portal vein (PV) pressure. We performed unroofing of the hepatic cyst and cauterization of the cyst wall on the hepatic side. Angiography was performed before and after unroofing of the liver cysts, and IVC stenosis release was confirmed. IVC pressure measured at the peripheral side of the stenosis and PV pressures were continuously measured during surgery and were confirmed to have decreased during the opening of the liver cysts. The patient had a good postoperative course and was discharged on the 10th postoperative day. No recurrence was observed 6 months postoperatively.

**Conclusions:**

Cyst unroofing surgery using angiography in a hybrid operating room is a useful treatment for deep hepatic lesions in that vascular stenosis improvement can be intraoperatively confirmed. Moreover, in cases wherein the cyst compresses the vasculature, intraoperative monitoring of IVC and PV pressures can be used to prove that the liver cyst is hemodynamically involved.

## Background

Liver cysts are a common disease in which patients are often asymptomatic. However, multiple or enlarged cysts can cause symptoms, such as abdominal pain and multiple organ compression; nevertheless, these are amenable to treatment. Liver cysts causing systemic symptoms due to hemodynamic disturbances of the inferior vena cava (IVC) or portal vein (PV) are very rare, with only a few reports to date. Liver cyst treatment includes medical therapy, such as percutaneous drainage and sclerotherapy with alcohol or minocycline injection into the cyst; however, recurrence cannot be avoided in these therapies [1]. Surgical treatment is the only curative option for hepatic cysts, and cystotomy and cauterization are widely performed as the first choice. General fenestration surgery is often performed for cysts on the hepatic surface of the anterior part of the liver [2–7]. Nevertheless, the reliable and effective treatment of such cases of vascular stenosis has not yet been established. Hybrid operating rooms have been used in a variety of surgical procedures. The advantages of the hybrid operating room include the ability to intraoperatively confirm the lesion and the therapeutic effect of the surgery [8]. Here, we report a case, wherein we performed and completed fenestration in a hybrid operating room with interventional radiology for a patient with portal hypertension and IVC and hepatic vein stenosis caused by multiple huge liver cysts.

## Case presentation

A 73-year-old male visited a previous doctor with a chief complaint of progressive leg edema and dyspnea on exertion 1 year prior. The patient had a history of spasmodic angina pectoris, midbrain infarction, and chronic renal failure. Computed tomography (CT) showed multiple hepatic cysts compressing the right lung, right side of the heart, and IVC. He underwent percutaneous aspiration for the largest cyst in segment eight of the liver; however, the leg edema did not improve, and the cyst flared up within a month. He was referred to our hospital for further investigation and treatment.

**Fig. 1:**
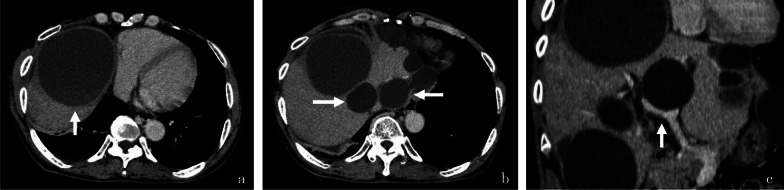
Computed tomography (CT) image taken at the first visit. The cyst is observed in the eighth segment of the liver and measures 13 cm in diameter; it is the largest of the cysts in this case. The cyst appears to have compressed the right side of the heart and the right thoracic cavity (**a**). The cysts are observed to be located dorsal to the liver on both sides of the inferior vena cava (IVC); they were thought to have caused the stenosis of the IVC (**b**). One of the cysts is seen to be in contact with the right and left branches of the portal vein; the right branch appears compressed and stenotic (**c**)

**Fig. 2:**
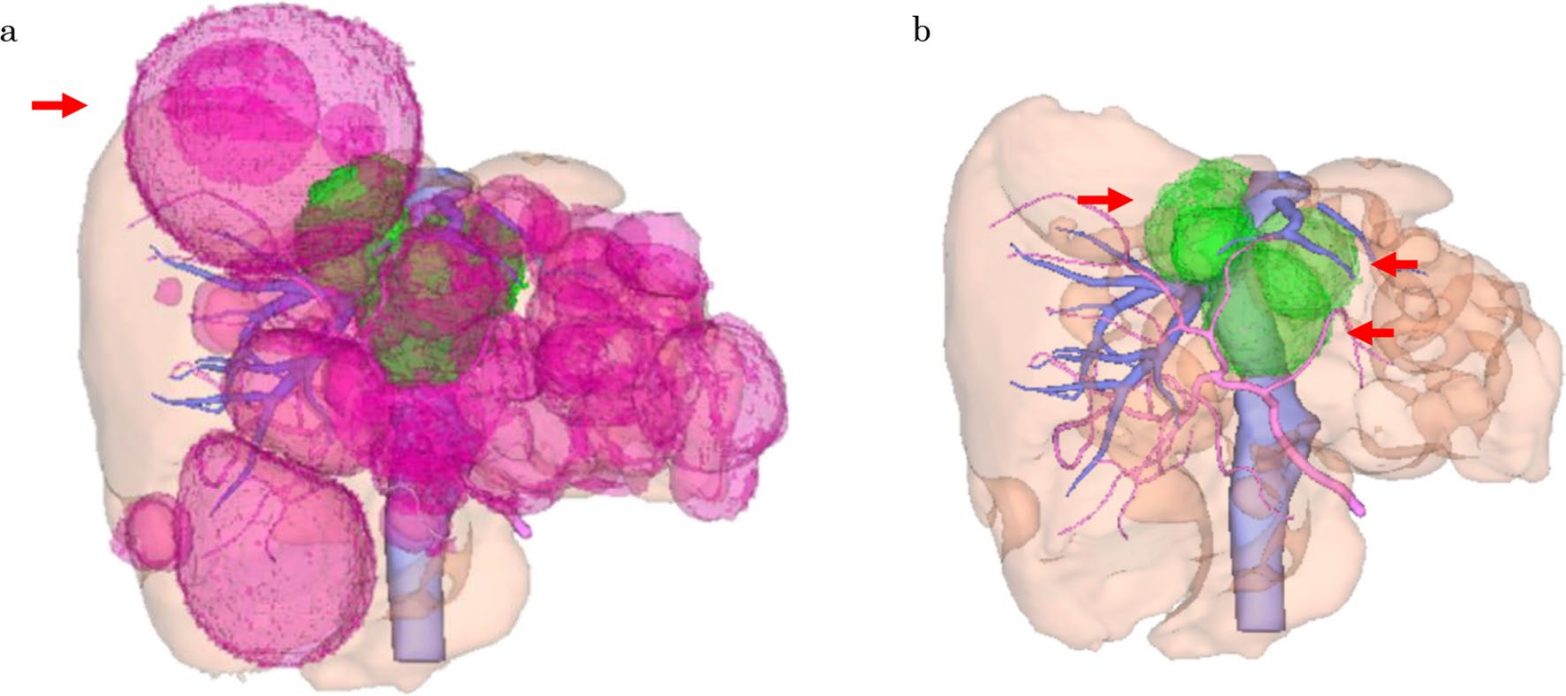
Three-dimensional reconstructed image of the liver. The hemodynamically involved cysts are indicated by arrows, respectively. **a** Shows all cysts, and the arrow indicates the largest lesion in segment eight. **b** Shows cysts on both sides of the IVC and near the portal bifurcation in green (arrowheads)

**Fig. 3:**
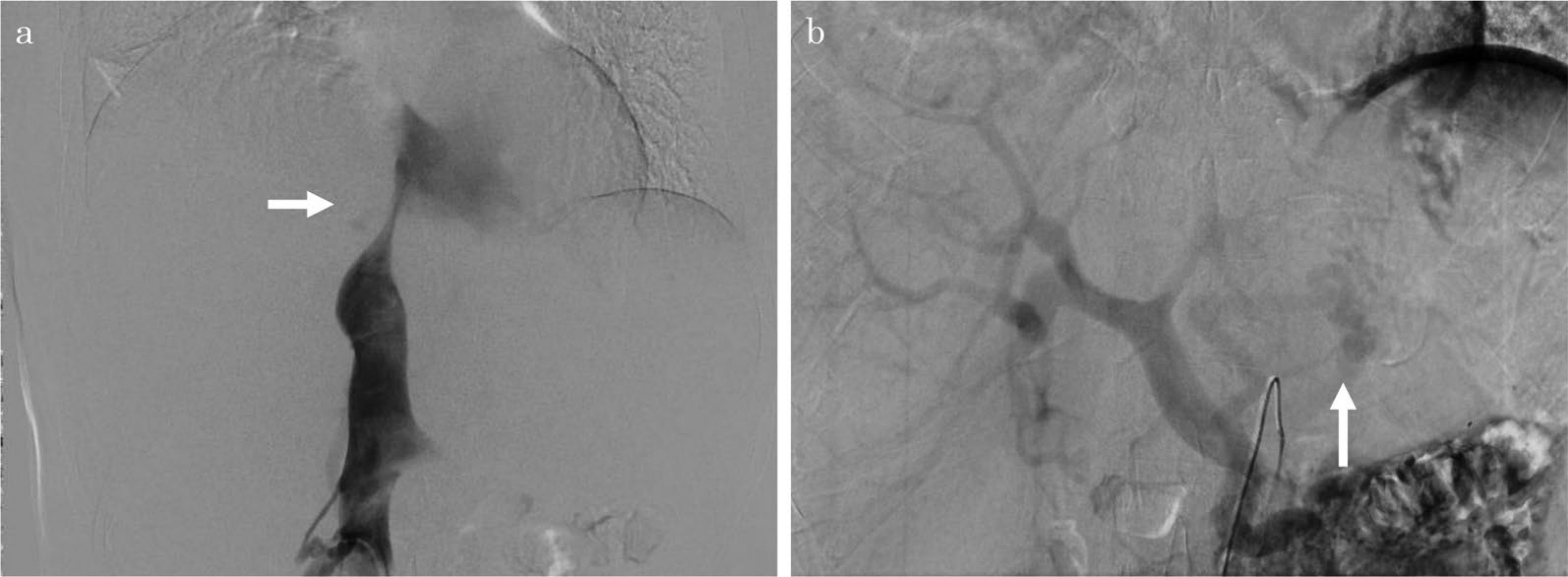
Preoperative angiography. Angiography confirms stenosis of the inferior vena cava (IVC) preoperatively (**a**). Angiography of the superior mesenteric artery reveals a left gastric vein–left renal vein shunt, although the portal veins enhanced the antegrade flow (**b**)

Blood tests at the time of our visit revealed no significant findings other than mildly decreased renal function (Table 1). CT showed multiple liver cysts, with the largest cyst in segment eight measuring 13 cm in diameter and compressing the right side of the heart and the right thoracic cavity (Fig. 1a). The IVC was bilaterally compressed by multiple liver cysts in the caudal lobe (Fig. 1b), implying blood flow obstruction. The cysts were also present in the hilar region of the liver, compressing the portal bifurcation and causing stenosis of the right branch of the portal vein (Figs. 1c, 2a, b). Angiography showed that the IVC was markedly stenotic, with an 11-mmHg pressure gradient on the central and peripheral sides of the IVC stenosis (Fig. 3a). Furthermore, superior mesenteric artery angiography showed that the portal vein enhanced the antegrade flow; however, left gastric vein–left renal vein shunt developed (Fig. 3b). No intestinal edema was found on imaging studies. Considering these imaging findings, pre- or post-hepatic mild portal hypertension (PH) may have occurred with the IVC compression.

**Table 1:** Laboratory findings before surgery

Hematology	
White blood cell	5.21 10^3^/µL
Hemoglobin	13.9 g/dL
Platelet	157 10^3^/µL
Biochemistry	
Aspartate aminotransferase	32 U/L
Alanine aminotransferase	28 U/L
Total bilirubin	1.7 mg/dL
Alkaline phosphatase	101 U/L
γ-Glutamyl transpeptidase	93 U/L
Blood urea nitrogen	22 mg/dL
Creatinine	1.22 mg/dL
Blood ammonia	19 µg/dL
Coagulation	
Prothrombin percentage activity	79%
International normalized ratio of prothrombin time	1.13
Carcinoembryonic antigen	3.9 ng/mL
α-Fetoprotein	2.9 ng/mL
Protein induced by vitamin K absence or antagonist II	19 mAU/mL
Other	
Indocyanine green 15-min retention	26.2
Child–Pugh score	A

Blood test findings on arrival at our hospital. Mildly decreased renal function was observed, but there were no other findings of note

Based on the above test results, we concluded that the IVC stenosis due to the liver cysts was the cause of the leg edema and dyspnea on exertion and hence decided to treat the liver cysts with fenestration. To confirm the hemodynamic status of the IVC, intraoperative angiography with CT during hepatic arteriography, CT during arterial portography, and angiography were performed in the hybrid operating room to allow IVC and PV angiography before and after liver cyst fenestration. Moreover, portal vein pressure (PVP) and central vein pressure (CVP) were continuously monitored intraoperatively as indices of hemodynamics in relation to the release of IVC stenosis. Furthermore, if the above methods did not improve the IVC stenosis, we were prepared for the dissection of the hepatic caudate lobe from the IVC and the placement of a metallic stent in the vessel.

**Fig. 4:**
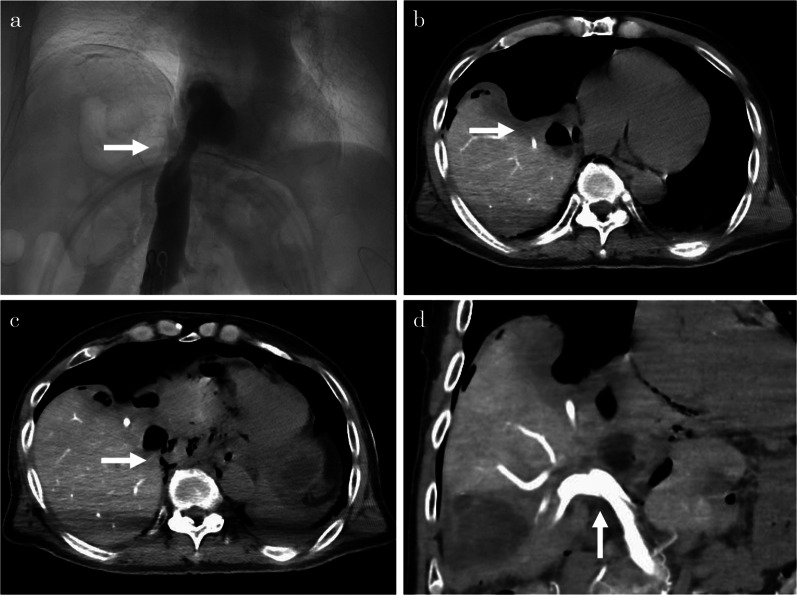
Intraoperative imaging studies. Angiography confirms that the stenosis of the inferior vena cava (IVC) was resolved after cyst fenestration (**a**). Computed tomography confirms that the cysts decompressed the right heart and that cyst fenestration released the IVC stenosis (**b**, **c**). Computed tomography arterial portography confirms that the stenosis of the portal vein had improved (**d**)

**Fig. 5:**
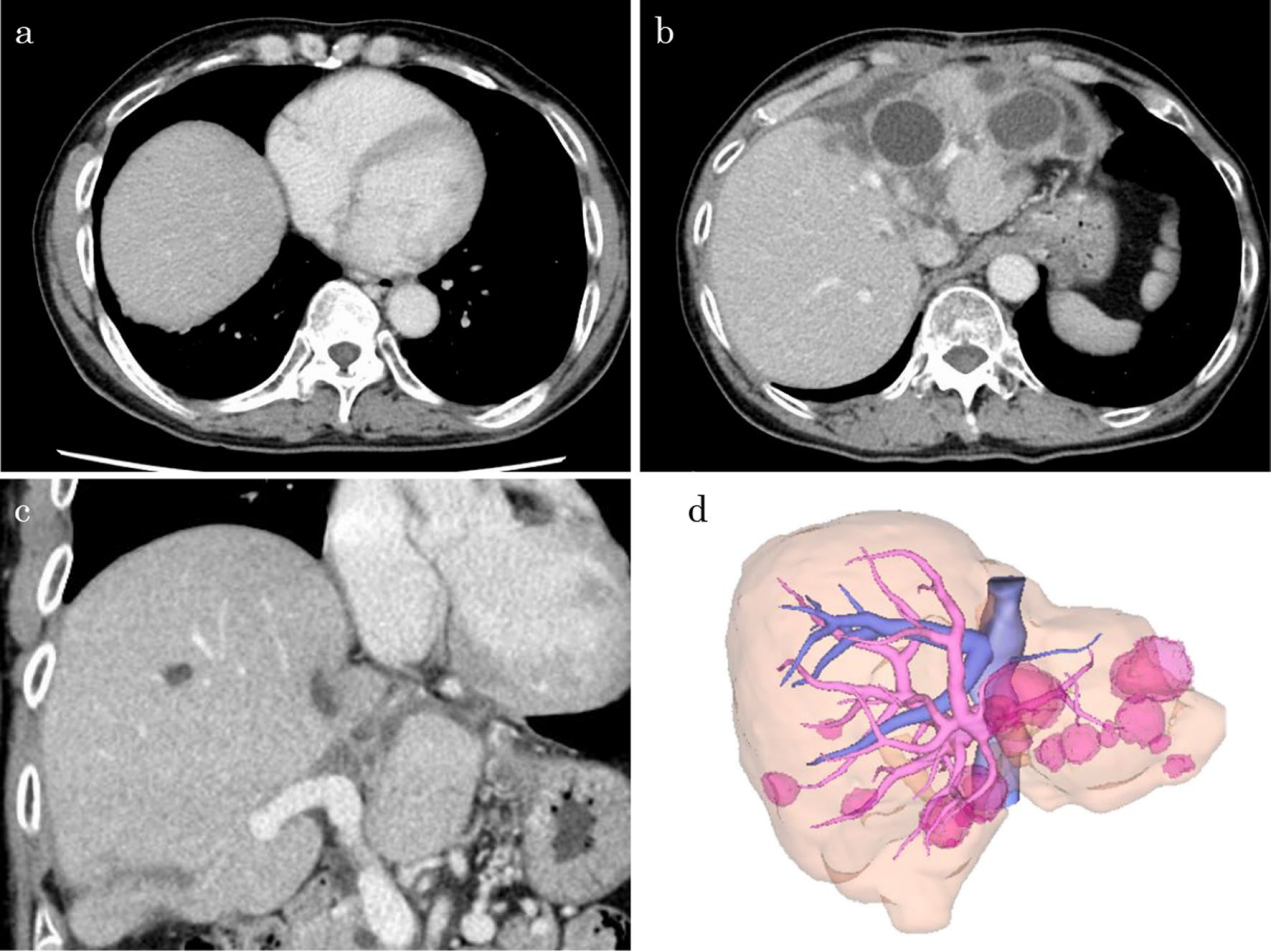
Computed tomography performed 6 months after the surgery reveals no recurrence of the right heart decompression (**a**), inferior vena cava stenosis (**b**), or portal vein stenosis (**c**). The 3D reconstructed image shows the disappearance of the cyst near the IVC and RHV (**d**)

The surgery was performed in a supine position under general anesthesia. To monitor PV pressure, a 15-Fr intravascular catheter (ANTHRON® P-U CATHETER; TORAY Medical, Japan) was inserted and fixed peripherally in the jejunum vein. CVP was defined as the pressure measured at the peripheral side of the IVC stenosis and measured by inserting a sensor through the right femoral vein. After mobilization of the left lobe of the liver, we fenestrated the largest lesion in segment eight of the liver. The CVP did not change before and after the fenestration of this lesion. After mobilization of the right lobe, the lesions on both sides of the IVC were identified under ultrasonographic guidance, and the cysts were fenestrated on the left and right sides of the IVC. Before and after fenestration, the CVP decreased from 5 to 1 mmHg, and the PVP decreased from 22 to 17 mmHg. The intraoperative changes in CVP and PVP are summarized in Table 2. Angiography findings and improvement of CVP confirmed that IVC stenosis was released after fenestration (Fig. 4a). Intraoperative CT findings also confirmed that the cysts that were causing the stenosis had been fenestrated (Fig. 4b-d). The cyst fundus was cauterized using a bipolar device, and a leak test was performed from the gallbladder duct to confirm that there was no bile leakage, finally completing the surgery. The operation time was 334 min, and the amount of bleeding during the surgery was 767 mL. The patient was discharged on the 10th postoperative day. His weight markedly decreased from 63.0 kg just before the surgery to 58.8 kg at the time of discharge. Six months after the surgery, the CT scan showed no obvious recurrence of the cysts (Fig. 5a-c). No leg edema recurrence or weight gain was observed.

**Table 2:** Intraoperative changes in CVP (IVCP) and PVP

Event	*IVCP (mmHg)	PVP (mmHg)
Start measuring	5	21
Fenestration of cyst		
Segment eight	10–8	24
Left side of the IVC	8–5	25
Right side of the IVC	5–5	22–19
Portal bifurcation	5–5	19–17
End of surgery	1	17

Intraoperative changes in central vein pressure (CVP) and portal vein pressure (PVP). After the measurement was started, CVP was increased by intraoperative infusion. After opening each cyst, CVP gradually decreased, especially when the cysts near the IVC were opened, CVP decreased significantly. PVP decreased when the cyst on the right side of the IVC was opened

*IVCP* inferior vena cava pressure, *PVP* portal vein pressure, *IVC* inferior vena cava

*CVP is the pressure measured at the peripheral side of the IVC stenosis. The sensor was inserted through the right femoral vein

## Discussion

To the best of our knowledge, this is the first report on the intraoperative confirmation of the surgical efficacy and successful completion of an operation for a complex condition under the combined use of multiple monitors and investigations in a hybrid operating room. The use of hybrid operating rooms in abdominal surgery is not yet commonly implemented, although there have been reports of such cases, including Furukawa et al.'s surgery for median arcuate ligament syndrome [9] and Soga et al.'s surgery for intra-abdominal bleeding due to trauma [10]. The use of the hybrid operating room offers several advantages, such as diagnostic imaging through angiography, intravascular pressure measurement, and lesion localization through intraoperative CT.

Liver cysts are a common liver lesion with an incidence of 0.17–4.75% [1]. Regardless of the number of cysts, most cases are asymptomatic with small cyst diameters, and few cases are amenable to treatment. By contrast, if there are any symptoms, treatment is indicated. A large liver cyst, despite being singular, may be perceived as abdominal distension, and if it drains the IVC or intrahepatic bile duct, it may present with symptoms, such as edema and jaundice [1, 4, 11]. Multiple liver cysts, including Caroli's disease, could reportedly present with clinical symptoms, such as liver failure and PH [12]. Liver cysts are also more common in females, with a male-to-female ratio of 1:1.5, and with the prevalence of symptoms likewise being higher among females [13]. Sanfelippo et al. have summarized the epidemiology of patients with liver cysts and reported that approximately 15% of patients with liver cysts are symptomatic [14].

The diagnosis of liver cysts is mainly based on imaging studies, such as ultrasonography, CT, and magnetic resonance imaging. Depending on the location of the lesion, interventional radiology or endoscopic retrograde cholangiopancreatography may be used to evaluate vascular and bile duct drainage and stenosis. The lesions located in the hilar or dorsal region up to the fourth/eighth segments are difficult to approach from the body surface; therefore, puncture drainage, effective therapy, and laparoscopic surgery cannot be performed.

Symptomatic liver cysts are amenable to treatment. The basic treatment involves drainage of the cyst contents and cauterization of the cyst wall. This can be performed by percutaneous cystocentesis, laparotomy fenestration, or laparoscopy. For multiple liver cysts or large lesions, hepatectomy, including the cysts, may be performed. If the lesions involve the entire liver, liver transplantation could be a last resort [1, 14, 15]. Additionally, for percutaneous treatment, sclerotherapy is performed, in which a drain catheter is placed in the cyst at the time of cyst puncture, and drugs, such as minocycline, ethanol, and picibanil, are injected [16]. In this case, the patient had symptoms of leg edema and dyspnea. The differential diagnosis included cardiovascular diseases, such as right heart failure, impaired venous return, hypoalbuminemia, and lymphedema. These systemic conditions could be ruled out from blood and urine tests. The IVC stenosis due to the cyst was clear on imaging, and we considered this the cause of the lower leg edema.

Liver surface cysts are commonly treated with surgical fenestration and cauterization [1, 17]. If symptoms, such as dyspnea, are observed due to failure of the right side of the heart caused by impairment by a large cyst, similar to our case, drainage and fenestration of the lesion should be first performed. General cystotomy is often performed laparoscopically for minimally invasive procedures [5–7, 17]. However, in our case, we recognized preoperatively that the caudal lobe lesion was involved in the IVC and right hepatic vein (RHV) stenosis, and we needed to perform reliable fenestration and drainage for the dorsal lesion as well. Therefore, we chose to perform a laparotomy.

The other treatment for vascular stenosis is endovascular stenting. It is an option for older adult patients who cannot undergo surgery and has the advantage of being less invasive. However, in this case, the stent was not used because the stenosis was not only in the IVC, but also in the RHV, and it was technically difficult to place the stent at the same time. In the preoperative evaluation, we suspected the possibility of post-hepatic PH due to impaired RHV and IVC outflow, and it was necessary to avoid blocking RHV outflow with a stent in the IVC.

In this case, the most important preoperative concern was to accurately identify the cyst responsible for the disease state and ensure that the disease would improve by fenestrating the cyst. To achieve this purpose, it is essential to evaluate the effect of treatment using multiple intraoperative monitors.

The choice of laparotomy made it possible to intraoperatively monitor the PVP and CVP. In general, liver cysts rarely cause PH [18]. In our case, the RHV outflow tract was stenotic because of liver cysts near the IVC, which led to PH. Intraoperatively, we were able to confirm that the PVP decreased from 22 to 19 mmHg before and after the opening of the cyst, which caused the RHV stenosis. This supported the diagnosis that RHV compression by the liver cyst was the cause of PH.

In addition, we were able to use a hybrid operating room with intraoperative IVR to confirm the target lesion. Although it is possible to locate cysts intraoperatively using echocardiography, it is sometimes challenging to visualize cysts around the IVC or on the dorsal side of the liver. Furthermore, if there is an image of IVC stenosis due to a cyst, the hybrid operating room has the advantage of intraoperative confirmation, especially after opening the cyst, such that the IVC stenosis can be released in situ. The disadvantage is that the operation time becomes inevitably longer owing to the need to move the operating table and secure a clean field for intraoperative CT. However, since we have been successful in performing the surgery while confirming reliable results, we consider this extension of the surgery time to be acceptable.

The recurrence rate after surgical treatment of liver cysts is reportedly 0–20% [1]. To prevent recurrence, reliable fenestration of the target lesion is required intraoperatively. Intraoperative CVP or PVP measurement and imaging studies with angiography and CT quantitatively and qualitatively confirm the effectiveness of surgery, which allows for reliable treatment. Ultimately, this is the greatest advantage of performing surgery for liver cysts in a hybrid operating room.

## Conclusions

In the present case, we performed preoperative planning and hybrid surgical fenestration for symptomatic liver cysts with good results. The use of a hybrid operating room for surgical fenestration is useful for deep liver cystic lesions that cause vascular drainage or stenosis. In addition, in cases wherein the cyst is compressing the vasculature, intraoperative monitoring of the IVC and PV pressures can be used to prove that the liver cyst is hemodynamically involved.

## Data Availability

The data that support the findings of this study are available from the corresponding author, S.K., upon reasonable request.

## Abbreviations

CT: Computed tomography
IVC: Inferior vena cava
CVP: Central vein pressure
PV: Portal vein
PHV: Portal hypertension
PVP: Portal vein pressure
CVP: Central vein pressure
RHV: Right hepatic vein
